# Supplementary material for: Complexome profiling of the Chlamydomonas psb28 mutant reveals TEF5 as an early PSII assembly factor
Source: Plant Cell. 2025 Mar 17;37(6):koaf055. doi: 10.1093/plcell/koaf055 (PMC12164590; doi:10.1093/plcell/koaf055)
Supplement: koaf055_Supplementary_Data [file koaf055_supplementary_data.zip › psb28_interactive.zip!/plots/intensity/qualityPlots/peptides/SHKA1_Cre17.g726750.t1.2.html]

### Cre17.g726750.t1.2

Trivial name: SHKA1  
SeqCoverage [median]: 7.40  
Mass: 55.75 kDa  
  
 

| Sequence | MissedCleavage | Mass [Da] | wtIntensity | muIntensity | Score |
| --- | --- | --- | --- | --- | --- |
| AKLPALIEAVQR | 1 | 1307.79 | 0.00 | 740.60 | 36.85 |
| GVNNPIGVK | 0 | 896.51 | 0.00 | 410.50 | 53.55 |
| SASTETIGGVTLPAYR | 0 | 1621.83 | 0.00 | 627.32 | 65.23 |
|  |  |  | 0.0 | 1778.4 |  |
